# Study on the Determination of Flavor Value of Rice Based on Grid Iterative Search Swarm Optimization Support Vector Machine Model and Hyperspectral Imaging

**DOI:** 10.3390/s24144635

**Published:** 2024-07-17

**Authors:** Han Yang, Fuheng Qu, Yong Yang, Xiaofeng Li, Ping Wang, Sike Guo, Lu Wang

**Affiliations:** 1 College of Computer Science and Technology, Changchun University of Science and Technology, Changchun 130022, China; sheep0012@163.com (H.Y.); yy@cust.edu.cn (Y.Y.); 2 College of Software Engineering, Jilin Technology College of Electronic Information, Jilin 132021, China; 3 State Key Laboratory of Black Soils Conservation and Utilization, Northeast Institute of Geography and Agroecology, Chinese Academy of Sciences, Changchun 130102, China; lixiaofeng@iga.ac.cn; 4 Jalaid Banner National Modern Agricultural Industrial Park Management Center, Hinggan League 137600, China; hlbwangping@163.com (P.W.); byndgsk@163.com (S.G.); 18204826555@163.com (L.W.)

**Keywords:** hyperspectral imaging, rice flavor value, particle swarm optimization (PSO), non-destructive technique

## Abstract

In the field of rice processing and cultivation, it is crucial to adopt efficient, rapid and user-friendly techniques to detect the flavor values of various rice varieties. The conventional methods for flavor value assessment mainly rely on chemical analysis and technical evaluation, which not only deplete the rice resources but also incur significant time and labor costs. In this study, hyperspectral imaging technology was utilized in combination with an improved Particle Swarm Optimization Support Vector Machine (PSO-SVM) algorithm, i.e., the Grid Iterative Search Particle Swarm Optimization Support Vector Machine (GISPSO-SVM) algorithm, introducing a new non-destructive technique to determine the flavor value of rice. The method captures the hyperspectral feature data of different rice varieties through image acquisition, preprocessing and feature extraction, and then uses these features to train a model using an optimized machine learning algorithm. The results show that the introduction of GIS algorithms in a PSO-optimized SVM is very effective and can improve the parameter finding ability. In terms of flavor value prediction accuracy, the Principal Component Analysis (PCA) combined with the GISPSO-SVM algorithm achieved 96% accuracy, which was higher than the 93% of the Competitive Adaptive Weighted Sampling (CARS) algorithm. And the introduction of the GIS algorithm in different feature selection can improve the accuracy to different degrees. This novel approach helps to evaluate the flavor values of new rice varieties non-destructively and provides a new perspective for future rice flavor value detection methods.

## 1. Introduction

Rice is a globally important food crop that provides the nutrition for a large portion of the world’s population. Rice is a staple food in many Asian countries and is rich in carbohydrates, vitamins and minerals. The concept of taste value is an evaluation of the texture and flavor of a food or beverage, including attributes such as sweetness, saltiness, acidity and bitterness. The taste value is usually quantified in whole numbers, using a numerical or hierarchical spectrum to indicate different intensities or perceptions of flavor.

With the improvement in living standards and the pursuit of a good quality of life, people are demanding better food quality and more flavor. Therefore, the importance of rice flavor values is increasingly recognized [1]. Determining the flavor profile of various rice varieties in a quick and easy manner is important for the advancement of agricultural practices. The traditional methods for assessing rice flavor include sensory evaluation and chemical analysis techniques. Sensory evaluation is mainly classified into appearance, texture and mouthfeel assessment involving the physiological attributes, hardness, viscosity and flavor of rice. In addition, swelling and water absorption tests involve cooking rice and observing its swelling and moisture absorption [2]. The chemical analysis techniques include the following:

1. Moisture content is assessed, usually by using the drying method or via titration. The drying method quantifies the moisture loss by drying the rice samples at a fixed temperature until a constant mass is reached. In contrast, the titration method uses an instrument such as a gas chromatograph to determine the moisture content by measuring the volume of water vapor [3,4].
2. Starch content test: Starch is the main component of rice and has a great influence on the flavor and taste of rice. A common method for determining the starch content is iodine titration. This technique quantifies the starch content by placing a rice sample in an iodine solution and observing the color change of the complex formed between starch and iodine [5].
3. Protein content testing: Protein is an important component of rice and plays an important role in the flavor and nutritional composition of rice. Protein content is usually assessed using the Kjeldahl method and Near Infrared Spectroscopy (NIRS). The Kjeldahl method assesses the protein content by quantifying the amino acid content of the rice samples after acid digestion and distillation. In contrast, NIRS uses a near-infrared spectrometer to examine the rice samples and determine the protein content by analyzing the spectral features [6,7].
4. Determination of the amino acid content: Amino acids are important nutrients in rice and have a significant impact on the flavor and nutritional value of rice. The quantification of the amino acid content is usually achieved via high-performance liquid chromatography (HPLC) and gas chromatography (GC). These analytical techniques involve extracting and separating the components in a rice sample and then determining the concentration of amino acids using specialized detectors [8,9].
5. Analysis of the aroma components: Aroma plays a vital role in the taste of rice. In order to analyze the aroma components, techniques such as gas chromatography–mass spectrometry (GC-MS) and odor assessment methods are commonly used. GC-MS determines the aromatic composition of rice by separating and characterizing the volatile components in the sample [10].

The odor assessment method requires the sensory analysis and characterization of the odor of rice samples by trained odor assessors. This sensory method, while valuable, is prone to subjective bias and may lead to inaccurate quantification and imprecise chemical composition analysis. Chemical analysis methods, while detailed, require skilled professionals, are time consuming and laborious, and sometimes cause damage to the rice samples. Therefore, there is an urgent need for a method that can quickly, efficiently and accurately characterize the various rice flavors.

Hyperspectral imaging provides a solution by providing continuous data in multiple spectral bands in the visible and infrared ranges. This imaging technique captures more detailed spectral information than conventional color imaging or human vision by continuously collecting narrow-band data in a specific spectral range and measuring the spectral reflectance or radiance of each pixel. As a result, hyperspectral images can detect the subtle variations and spectral characteristics of target materials, making it easy to analyze and distinguish between various substances and objects [11,12,13,14]. At the same time, the nature of hyperspectral data makes data processing and analysis very complex, requiring the use of dimensionality reduction or feature selection techniques to reduce the data dimensionality, as well as effective pre-processing and variable selection methods to extract the useful information and remove irrelevant or noisy data. The magnitude of the flavor value of rice, i.e., the texture of rice, is determined by the content of straight-chain starch, and the moisture and protein in the rice [15]. The content varies between different types of rice, which leads to the differences in reflectance in the hyperspectral bands. In this study, different varieties of processed rice were selected as the focus of the study. Using the methods discussed earlier, hyperspectral data were collected for each rice variety in order to assess its unique flavor profile. The analysis consisted of correlating the reflectance observed in each band of the hyperspectral image with the corresponding flavor values. This approach enables the effective detection of flavor values of rice through the subtle analysis of the hyperspectral data.

Although the traditional PSO algorithm requires fewer parameters and can effectively improve the computational efficiency, it has the disadvantages of being easy to fall into the local optimum and an unstable convergence speed; especially when the search range is large, the disadvantages are more obvious [16]. In this paper, by combining with other algorithms in the form of a certain degree of reduction, the PSO algorithm needs to find the paradigm dimension space, effectively improving the PSO algorithm’s ability to search for the optimal solution, thereby reducing the time cost of the search for the optimal solution over a wide range.

## 2. Materials and Methods

### 2.1. Data Sources

In this study, 40 rice varieties grown in the National Modern Agricultural Industrial Park of Zhalite Banner, Xing’an League, Inner Mongolia Autonomous Region, were selected for the study. According to the principle of random allocation, samples of Tianyu 9516, JINONGDA 673 and CKF 8 were collected. Prior to the experiment, the collected rice underwent a series of preparation processes: threshing, hulling and milling. Subsequently, immature and small-sized grains were discarded, leaving only full-sized grains for further analysis. These kernels were then scanned and evaluated via hyperspectral imaging to determine their flavor values. For each of the 40 rice varieties, three flavor assessments were made using the Rice Flavor Meter. The average of these three measurements was recorded as a parameter for the determined flavor value for each rice variety.

### 2.2. Hyperspectral Imaging Systems and Data Acquisition

A hyperspectral imaging system with a spectral range of 400 to 1000 nm was utilized to acquire the reflectance mode images of processed rice. The system consists of a charge-coupled device (CCD) camera, an imaging spectrometer (Pika XC2, Reson Inc., Bozeman, MT, USA), a zoom lens (XENOPLAN, F/1.4 FL23 mm, Schneider—KREUZNACH, Bad Kreuznach, Germany), an illumination unit (OSRAM, Munich, Germany)(a 4-lamp illumination system, 35 W per lamp, total input power 140 W, total radiant power about 5~7 W), and a computer with data acquisition control software (SpectrononPro software, Version 2.100, Fermi Resonance Inc., Bozeman, MT, USA). The imaging spectrometer has a spectral range of 400~1000 nm, a slit of 50 μm, a spectral resolution of 1.3 nm, and a spatial resolution of 0.15 mm/pixel. This comprehensive system facilitated the collection of hyperspectral images for 40 rice varieties, each represented by approximately 70 seeds across 462 spectral bands.

The hyperspectral instrument was first warmed up for 30 min to ensure its optimal performance. The acquisition parameters were then fine-tuned using the Spectral VIEW software, setting the exposure time to 20 ms. Its calibration was performed using the optical whiteboard provided with the system. This involved placing the lens cap on the hyperspectral lens to capture a full black spectral image and then capturing an image of the polytetrafluoroethylene (PTFE) optical whiteboard. These black and white images were used to calibrate the instrument, and the calibration equation was applied to derive a calibration reference value. This calibration process is critical to ensure accurate and reliable hyperspectral data acquisition, as described in Equation (1).

(1) $$R_{\lambda }=\frac{I_{\lambda }-H_{\lambda }}{B_{\lambda }-H_{\lambda }}$$

In Equation (1), $R_{\lambda }$ represents the calibrated sample image data, $H_{\lambda }$ denotes the all-black image acquisition data, $B_{\lambda }$ is the all-white image acquisition data, and $I_{\lambda }$ signifies the original sample image acquisition data.

To ensure the optimal image quality, the integration time was carefully adjusted to avoid overexposure. A dark current was then acquired to further optimize the image quality, and the image aspect ratio was adjusted for accurate rendering.

**Figure 1:**
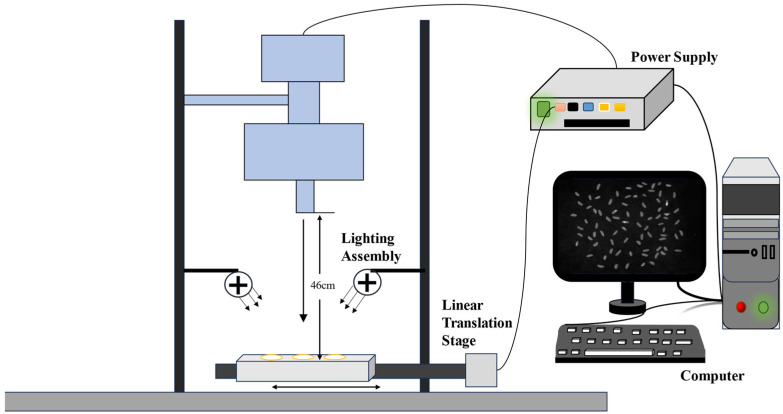
Schematic diagram of hyperspectral acquisition process.

During the scanning process, the rice grains were evenly distributed across the plates, which were covered with light-absorbing material to minimize reflections and ensure uniform illumination conditions. The hyperspectral information of the rice grains on each plate was then systematically captured as the linear stage moved within the imaging system, resulting in a full scan of all the samples. The process is shown in Figure 1. This meticulous setup and procedure ensured that high-quality hyperspectral data were obtained for the subsequent analysis.

### 2.3. Image Processing and Spectral Extraction

**Figure 2:**
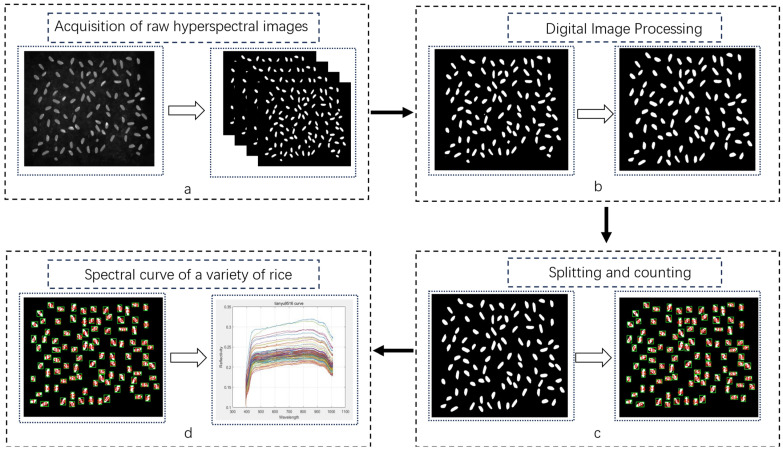
Hyperspectral image processing and curve plotting: (**a**) image binarization; (**b**) Remove blocks with fewer pixel values; (**c**) Number each rice kernel in order from left to right from top to bottom; (**d**) Extracted hyperspectral image, each curve represents a grain of rice.

Rice grains, which have an approximately elliptical shape, have a good transmittance, which minimizes the effect of the light source reflections on the acquired hyperspectral images. However, due to the presence of dust and other impurities on the light-absorbing material, the image needed to be segmented to effectively separate the rice grains. For this purpose, an Otsu threshold segmentation method was used with morphological erosion of each connected region. This method helps to eliminate extraneous fragments smaller than a specified pixel value in a single connected region. The specific process is as follows:

1. Using the color thresholding method, the rice hyperspectral image in a single band is binarized, and the unwanted background pixels are set to zero (black) to obtain the image shown in Figure 2a. The 188-band rice reflectance image was chosen for illustration because it provides the best clarity in depicting rice grains.
2. The binarized image is eroded to remove small blocks with pixel values less than 500 in any single connected region, as shown in Figure 2b. The binarized image is then processed to remove all the pixels in the image.
3. The processed hyperspectral images are numbered sequentially from top to bottom and left to right. Each threshold range is marked with a green rectangle, and the corresponding number is displayed on a graph; the result is shown in Figure 2c. This step helps in the subsequent matching of odor values.
4. The white areas in Figure 2c are the study areas. When acquiring hyperspectral data, all 462 bands were masked and all non-zero pixel values for the bands within each threshold range were averaged. The resulting average value represents the spectral reflectance of rice in that threshold range, resulting in the hyperspectral profile image shown in Figure 2d.

This approach ensures accurate segmentation and analysis of the hyperspectral images for the precise correlation with the rice flavor values.

### 2.4. Spectral Curve of a Variety of Rice

**Figure 3:**
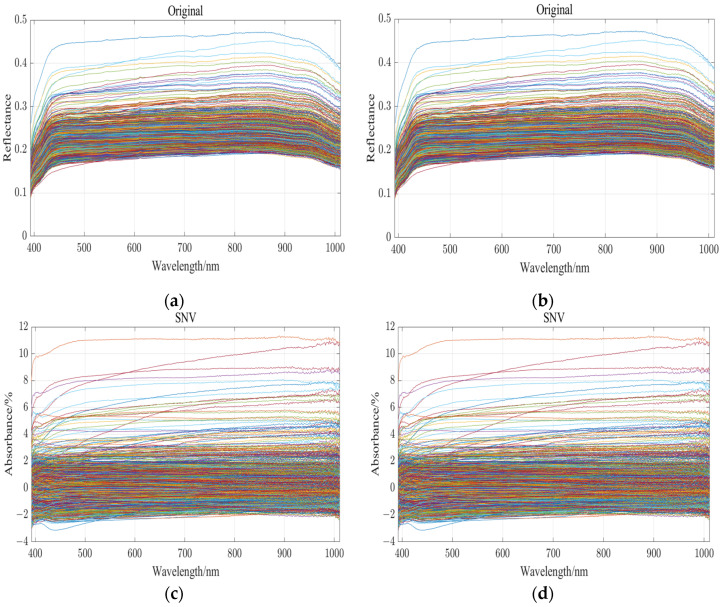
Spectral curve preprocessing: (**a**) original spectral curve; (**b**) spectral curve after smoothing; (**c**) spectral curve after SNV; (**d**) spectral curve after MSC. Each curve represents a grain of rice.

During the acquisition of hyperspectral data, instrumental and environmental factors can affect the quality and accuracy of the data to varying degrees. To mitigate these effects and improve the reliability of the data, the raw images were subjected to pre-processing techniques including Multiplicative Scatter Correction (MSC) [17], smoothing [18] and Standard Normal Variation (SNV) [19]. These methods are designed to reduce the impact of extraneous variables on the data [20]. Smoothing is a key preprocessing step in hyperspectral data analysis aimed at improving the signal-to-noise ratio. The technique effectively reduces a wide range of noise that may come from environmental factors such as instruments, air or other sources. Smoothing reduces the impact of these noise factors by averaging the data locally or globally. This process involves averaging the data points within a specified neighborhood or across the entire dataset to reduce the effects of outliers and random noise. As a result, the data become smoother, which simplifies further analysis and processing. Smoothing not only improves the quality of the data, but also helps to extract meaningful information, and is therefore an important step in the pre-preparation of the hyperspectral data for detailed examination and interpretation [21]. The Standard Normal Variate (SNV) transformation is a preprocessing technique used in hyperspectral data analysis to standardize each spectrum in a dataset. By adjusting the spectra to have a mean of zero and a standard deviation of one, SNV effectively reduces the intensity differences between the samples of different species. This normalization process makes the spectral data more homogeneous in terms of internal intensities, which is particularly beneficial when developing predictive models [22]. The results of using the four preprocessing techniques are shown in Figure 3. By applying these methods in concert, variations due to the internal and environmental factors within the instrument are greatly reduced and the effects of noise are mitigated, making the data more suitable for further analysis, modeling, and interpretation. To determine the optimal order in which to apply the three algorithms, we trained each algorithm independently on the same data model, and the results are shown in Table 1.

**Figure 4:**
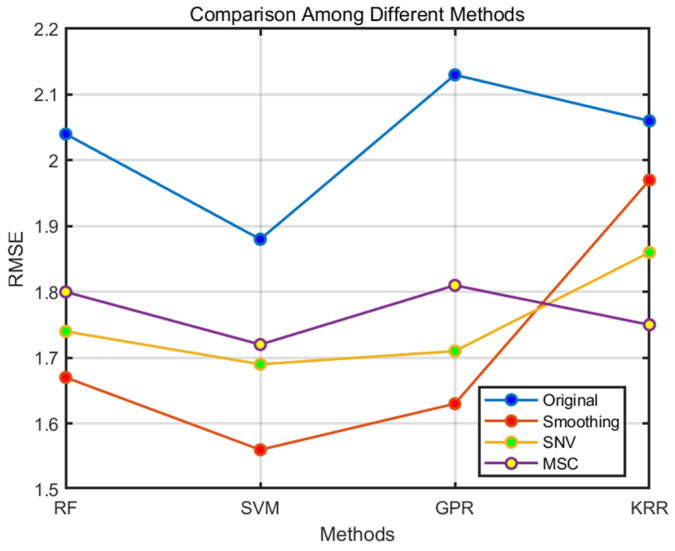
Comparison of different preprocessing methods.

As the data in Table 1 and Figure 4 show, the results of the four different preprocessing methods differ in terms of their model training efficiency. Notably, the combination of smoothing with the support vector machine (SVM) regression algorithm produced the most favorable results, achieving a root mean square error (RMSE) of 1.56. Overall, the smoothing method performed well in reducing the error in the prediction results compared to the other preprocessing methods. In addition, the SVM model outperformed the other regression models across all the data conditions, showing a stronger performance. In view of these findings, SVM was selected as the primary regression model for data analysis. Therefore, as a preprocessing step for the hyperspectral data, Multiplicative Scatter Correction (MSC), Standard Normal Variate (SNV) and smoothing were applied sequentially.

Given the abundance of data provided by hyperspectral imaging systems, as the number of bands used for model training increases, the distinctions between the bands become less pronounced, leading to lower values of the band attributes and longer model training times, which in turn adversely affects the accuracy of the model. To address this problem and reduce the amount of data, we used two methods: the Competitive Adaptive Reweighted Sampling (CARS) [23] and Principal Component Analysis (PCA) [24,25,26] to reduce the dimensionality. These methods were compared to the full 462-band dataset to determine a more efficient regression scheme.

**Figure 5:**
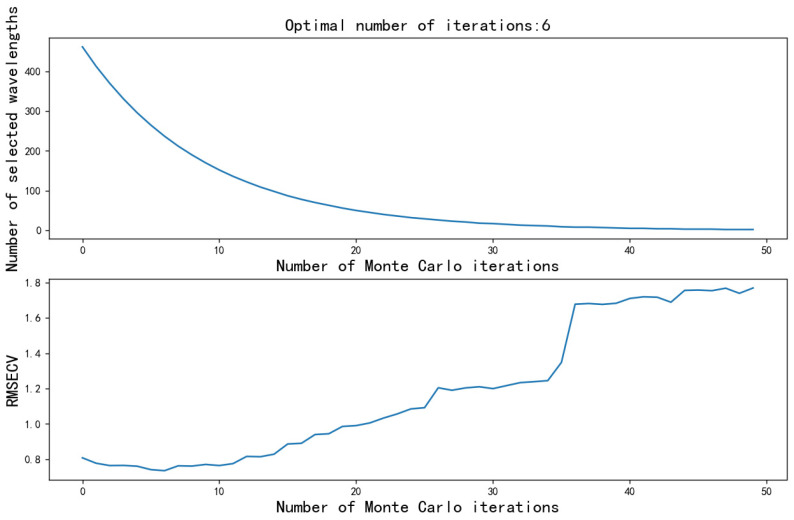
CARS algorithm results.

The Competitive Adaptive Weighted Sampling (CARS) algorithm is designed to identify characteristic bands in hyperspectral spectra. It uses a combination of partial least squares regression and Monte Carlo random sampling for the numerical analysis. Through cross-validation, CARS obtains sampling results with minimal cross-validated root mean square error (RMSECV). Inspired by the Darwinian principle of “survival of the fittest”, CARS introduces a variable selection algorithm to optimize the selection of spectral bands by iteratively improving the statistical information. This approach helps to efficiently identify the most informative bands, thus significantly improving the performance of hyperspectral data analysis [27,28]. Figure 5 shows the relationship between the number of Monte Carlo simulation iterations, the size of the cross-validation root mean square error (RMSECV) and the number of feature wavelengths selected. In the sixth iteration of the Monte Carlo process, the RMSECV of the algorithm reaches its lowest, and accordingly, 234 feature wavelengths were selected. This finding highlights the efficiency of the iterative process in optimizing the spectral features for the hyperspectral data analysis.

The Principal Component Analysis (PCA) algorithm is capable of handling a number of areas such as data visualization, dimensionality reduction, denoising and feature extraction. It has wide applicability in managing high-dimensional data and facilitating exploratory data analysis [29,30]. In this study, 10, 20 and 50 principal components were finally selected for evaluation, which demonstrates the versatility of PCA in optimizing the structure of the data to improve the results of the analysis.

In this study, Root Mean Square Error (RMSE) and accuracy metrics were used to assess the quality of the model’s prediction results. The root mean square error is calculated as follows:

$$RMSE=\sqrt{\frac{1}{n}{\sum \left(y_{i}-y_{j}\right)}^{2}}$$

where:

RMSE represents the root mean square error.

n is the number of observations or predicted values.

$y_{i}$ is the i th observed value.

$y_{j}$ is the j th predicted value.

Σ symbolizes the summation operation, aggregating across all observations or predicted values.

Additionally, the accuracy of the prediction results is determined by rounding the predicted values and comparing them with the actual observed values. The method for calculating the accuracy is as follows:

$$A\mathrm{ccuracy}=\frac{\mathrm{Number}\;\mathrm{of}\;\mathrm{Correct}\;\mathrm{Predictions}}{\mathrm{Total}\;\mathrm{Number}\;\mathrm{of}\;\mathrm{Predictions}}.$$

### 2.5. GISPSO Algorithm

The particle swarm optimization (PSO) algorithm enhances the parameter selection by simulating the search behavior and information exchange of particles in the parameter space. The algorithm has global search capability and strong convergence properties to find the optimal solution in a relatively short time.

Support vector machines (SVMs) excel at managing complex datasets and solving nonlinear regression challenges. However, their effectiveness in real-world applications depends on careful hyperparameter tuning. Determining the optimal combination of parameters is critical to improving the performance of SVMs [31].

PSO-SVM integrates the parameter optimization challenge of SVMs into the search for optimal parameter combinations, using a particle swarm optimization (PSO) algorithm to find the best solution in the parameter space. In this setup, each particle represents a potential parameter combination, and the particle swarm consists of multiple such particles. These particles are iteratively explored in the parameter space with the aim of discovering the best combination that yields the highest performance of the SVM model.

Given the complex nonlinear relationships that often occur in hyperspectral data, the kernel function of SVMs provides a flexible approach to solving these nonlinear problems. PSO enhances this process by identifying the most appropriate kernel function and its associated parameters to ensure a better fit to the data features. Since hyperspectral data contain a large amount of information, PSO’s powerful global search capability helps to find potential global optimal solutions, making it a powerful tool for improving the performance of SVM models in hyperspectral data analysis [27]. In the process of acquiring hyperspectral images, it is inevitable to encounter various noise points and interfering information, and an SVM itself has a certain anti-noise ability. Optimization of the SVM by using PSO can fine-tune the parameters of the SVM and select the best values to further enhance the noise immunity of the model. This optimization process ensures that the model is still effective in the presence of noise, thus improving its reliability and accuracy in analyzing hyperspectral data [32,33,34].

**Figure 6:**
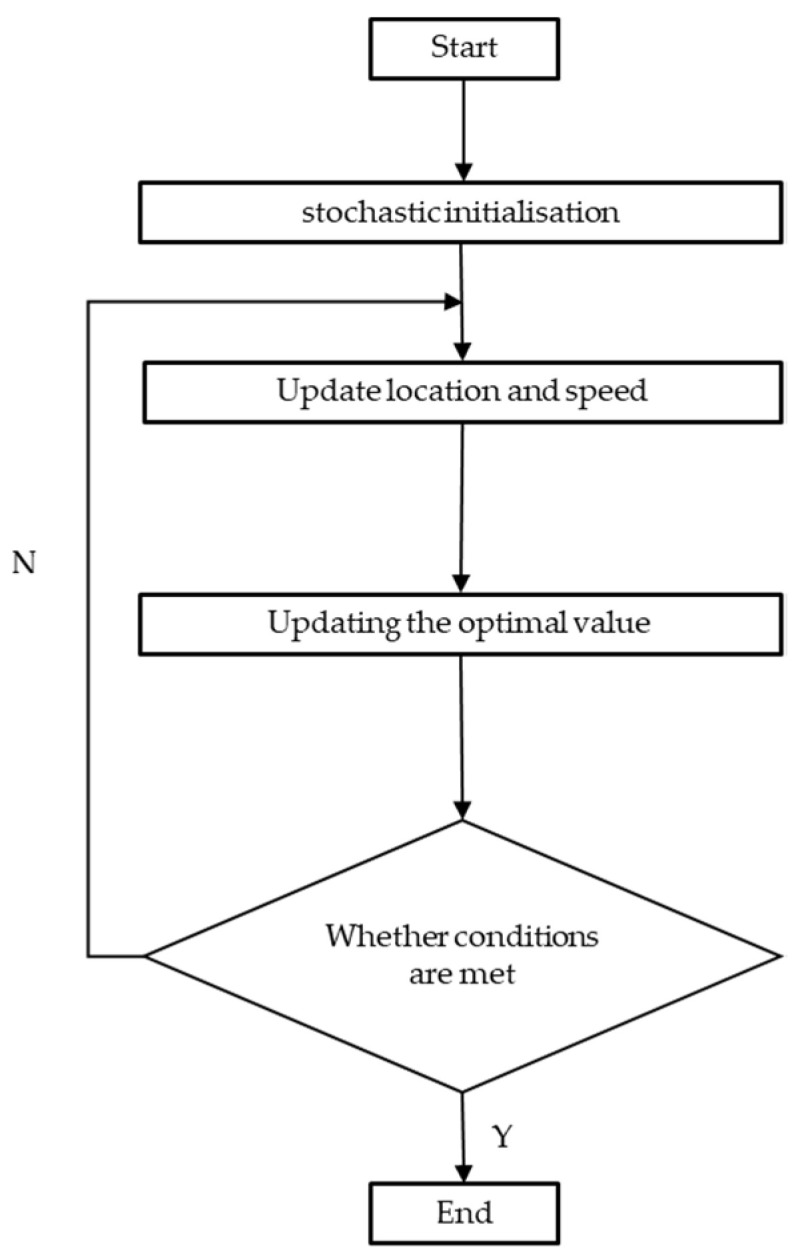
PSO optimization algorithm flow.

The flowchart of PSO is depicted in Figure 6.

In the particle swarm optimization algorithm (PSO), the formula for the position update is shown below:

For the dth dimension of the ith particle, the formula for the position update is:

$$\theta_{i,d}\left(t+1\right)=\theta_{i,d}\left(t\right)+v_{i,d}\left(t+1\right)$$

where,

$$v_{i,d}(t+1)=𝟉\cdot v_{i,d}(t)+c_{1}\cdot r_{1}\cdot \left({\hat{\theta }}_{i,d}(t)-\theta_{i,d}(t)\right)+c_{2}\cdot r_{2}\cdot \left({\hat{\theta }}_{g,d}(t)-\theta_{i,d}(t)\right)$$

$\theta_{i,d}(t)$ is the current position of particle i in dimension d.

$v_{i,d}(t)$ is the current velocity of particle i in dimension d.

𝟉 is the inertia weight, which is used to balance the direction of motion of the particles and generally takes a value between [0, 1].

$c_{1}$ and $c_{2}$ are acceleration coefficients representing the importance of individual and group experience, respectively, and generally take values between [0, 2].

$r_{1}$ and $r_{2}$ are random numbers, usually taking values between [0, 1].

${\hat{\theta }}_{i,d}(t)$ is the individual optimal position of particle i in dimension d.

${\hat{\theta }}_{g,d}(t)$ is the globally optimal position in the particle swarm.

The choice of parameters and formulas in particle swarm optimization (PSO) is tailored to the specific problem at hand and the chosen PSO variant. When optimizing a support vector machine (SVM) using PSO, the performance of the SVM is usually used as a fitness function, while the particle positions represent the parameters of the SVM, in particular, c (the regularization parameter), and g (the kernel function parameter). By interacting and exchanging information with neighboring particles, the particle swarm seeks the optimal hyperparameter configuration.

However, the inherent local search properties of PSO can sometimes lead to particles falling into local optima. This premature clustering towards the local optimum may prevent the swarm from discovering more favorable solutions, thus highlighting the limitations of the algorithm in effectively balancing exploration and exploitation [35].

The grid search (GS) algorithm for optimizing support vector machines (SVMs) consists of defining a range of values for the SVM hyperparameters and performing an exhaustive search within a predefined parameter space using a specific step size. This process involves testing various parameter combinations and evaluating the performance of each to determine the optimal set of hyperparameters. A significant advantage of this approach is the ability to accurately find the globally optimal solution, as it methodically explores all the possible combinations, thus avoiding falling into the error of local optimality. However, lattice search algorithms face challenges when dealing with large datasets or more complex scenarios. The exhaustive nature of the search leads to an increase in the number of grid points, which in turn significantly increases the computational time and cost. As a result, this approach is resource and time intensive and is less efficient in applications that require fast data processing or limited computational resources [36].

For scenarios involving large datasets, this study proposes a hybrid approach that exploits the advantages and mitigates the drawbacks of both the particle swarm optimization (PSO) algorithm and the grid search (GS) algorithm. The PSO algorithm, which is known for its low computational cost, has a tendency to converge to a local optimum, while the GS algorithm, despite its high computational requirements, is better at identifying the global optimum.

The proposed method, Grid Iterative Search Particle Swarm Optimization (GISPSO), employs large step intervals to determine an initial global optimum (denoted as P1) over a wide range of parameters of the SVM, starting with a broad GS. This step suggests that the true global optimum may lie in the vicinity of P1, but the coarse-grained nature of the initial search may not allow for the precise localization of the optimal parameters. Subsequently, the search range is narrowed down to the vicinity of P1 and the step size is reduced to obtain a finer GS. This process is repeated, gradually narrowing down the search range and the step size, until a predetermined target range is reached. At this point, PSO will accurately locate the global optimum within the reduced range.

**Figure 7:**
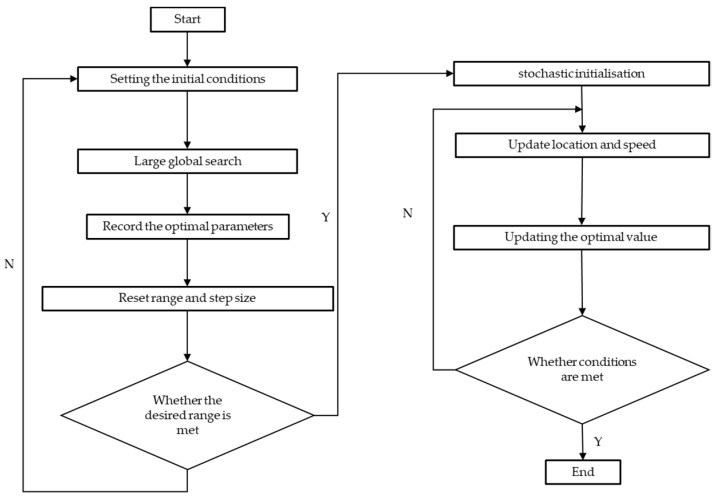
GISPSO algorithmic process.

GISPSO efficiently optimizes the SVM model to ensure fast and accurate determination of the global optimum in large and complex datasets. This approach circumvents the limitation of the PSO algorithm that local optimization may occur during an extensive search and reduces the time and resource consumption of the GS algorithm. The procedure of GISPSO is shown in Figure 7.

## 3. Results and Discussion

After screening the relevant features using various feature selection algorithms, the regression model was applied to the dataset, which was divided into a training set and a test set in the ratio of 7:3. The test set was then evaluated using the model trained on the training set. The performance of the model in the test set was evaluated by calculating the root mean square error (RMSE) based on the actual flavor values in the test set. In addition, given that the predictions are continuous values and flavor values are usually integers, the predictions were rounded to the nearest integer. This rounding allows for direct comparison with observations and facilitates the calculation of the model’s accuracy. The results of the evaluation are presented in Table 2, from which the predictive performance and accuracy of the model in estimating flavor values can be seen.

**Figure 8:**
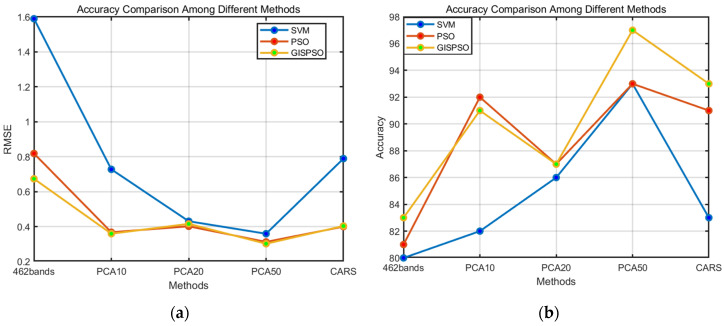
Result graph. (**a**) Comparison of RMSE for results of different methods; (**b**) comparison of accuracy for results of different methods.

**Figure 9:**
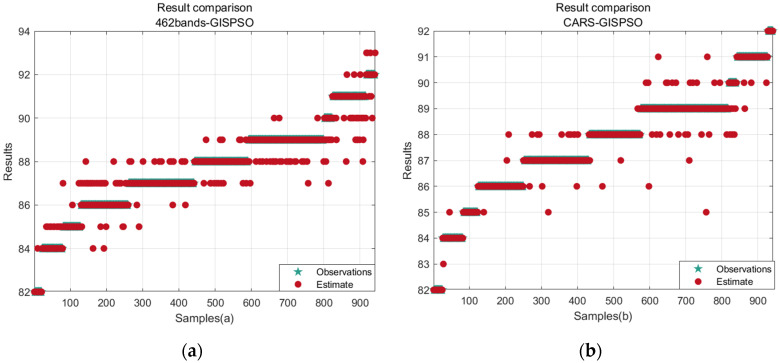

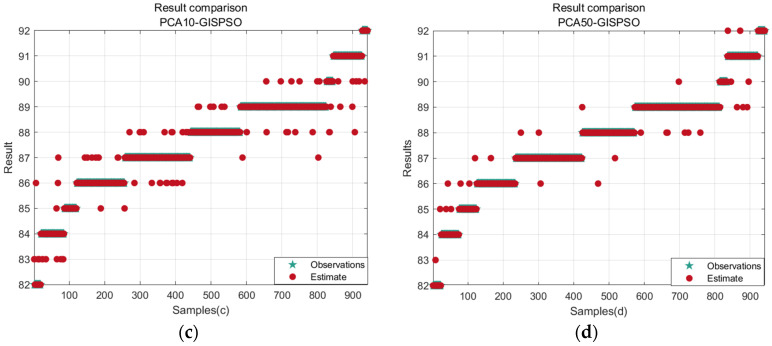
Comparison Scatterplot. (**a**) Full band optimization prediction result; (**b**) CARS feature selection prediction result; (**c**) PCA downscaling 10 principal component prediction result; (**d**) PCA downscaling 50 principal component prediction result.

Combining the corresponding statistical analyses in Table 2 and Figure 8 and Figure 9, it is obvious that the Grid Iterative Search Particle Swarm Optimization (GISPSO) algorithm effectively mitigates the limitations of the particle swarm optimization (PSO) algorithm, in particular its susceptibility to the influence of boundaries and local optimums. The GISPSO algorithm, by pre-determining a range of the near-optimal parameters, greatly reduces the randomness related to the direction and velocity of PSO particles in the optimization process and velocity-related randomness in the optimization process. This strategic approach allows for a more focused search within the near-optimal range, thus improving the accuracy of the optimization.

The performance gap between the PSO algorithm applied to full-band data and PCA50 processed data compared to the GISPSO algorithm highlights the efficacy of the GIS pre-search in circumventing the parameter constraints. The penalty factor c of SVM tends to increase while the kernel parameter g tends to decrease in cases involving larger datasets or more complex relationships. Table 3 details the optimization results for various parameter presets, where the parameter g reaches the preset minimum value of 0.1 for group 1, while the parameter c reaches the preset maximum values of 200 and 300 for groups 2 and 3, respectively. Within the preset parameter ranges of the PSO algorithm, the parameters are likely to hit the boundaries during the optimization search. This limitation, together with the predefined parameter ranges, re-limits the exploration of other parameter combinations and may lead to the premature termination of the algorithm and the misidentification of the optimal solution within the current parameter range as the combined optimal solution.

**Figure 10:**
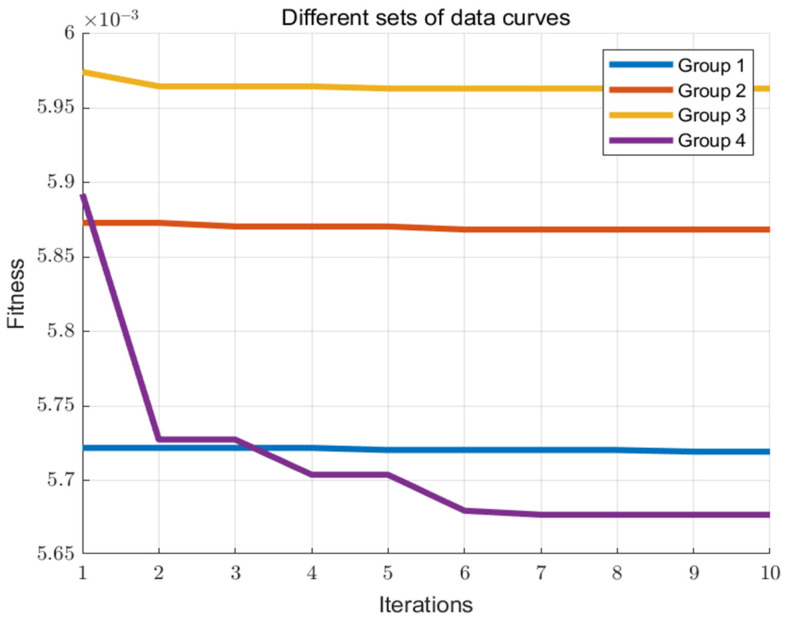
Fitness curves for different parameters.

When dealing with different problems, the parameter range settings, which are usually derived empirically, can have a significant impact on the final optimization results. Too narrow a range may limit the exploration, while too wide a range may lead to a significant expenditure of time and resources. As shown in Figure 10, the fitness curves for the first three sets of parameter ranges change very little and end the optimization search prematurely, with no further change in the fitness curves as the number of PSO iterations increases. This suggests that the algorithm’s parameter settings may not be optimally configured, thus limiting the effective exploration of the solution space, or that the algorithm has converged to a local or global optimum.

By pre-determining the optimal parameter ranges through the GIS algorithm prior to initiating a PSO search, the GISPSO method significantly reduces the likelihood of encountering a boundary optimum during the PSO process. This improvement narrows the search range and allows PSO to determine the global optimum parameters more accurately and quickly. As a result, this improves the efficiency of the algorithm and confidence in the results, while reducing the risk of converging to a local optimum.

SVM regression algorithms typically perform well on small sample datasets and rely heavily on support vectors during the optimization, which helps to determine the optimal regression hyperplane so that the model generalizes well even on small amounts of data. When the data dimension is high, SVM regression can map the data to a higher dimensional feature space through the kernel function, which better captures the complex nonlinear relationships. Also, SVM regression is typically faster to train than deep learning models and can be solved faster using efficient optimization algorithms. In contrast, deep learning models may require a large amount of data to avoid overfitting, especially in high-dimensional spaces, and deep learning models typically require more computational resources and time for large-scale gradient descent optimization. The PCA + SVM algorithms used in this paper usually perform well in high-dimensional feature space with less data because they can learn effective patterns on less data and are less prone to overfitting. PCA can provide the principal components of the data, which helps to understand the variations and the main features of the data; an SVM generates a model with a relatively simple structure, and the distribution of the support vectors helps to explain the formation of the decision boundaries and the classification. When the dataset size is small, PCA combined with SVM regression usually provides more stable and accurate prediction results; an SVM optimizes the regression curve via interval maximization, while PCA improves the algorithm’s processing efficiency and prediction speed through feature compression. Meanwhile, compared with complex deep learning models, PCA combined with SVM regression has relatively few key parameters, such as the number of principal components of PCA and the type of kernel function of the SVM. PCA also filters out some noise and redundant information, which makes it more robust than deep learning when dealing with generally noisy datasets. In this paper, we use the SVM algorithm to map the data to a higher dimensional feature space via the Gaussian kernel function, combined with the PCA feature processing method, which is able to deal with complex data patterns and nonlinear relationships, and has a better performance in this experimental data.

Baichuan Jin et al. [37] used near-infrared hyperspectral imaging combined with deep learning to identify the rice varieties. The ResNet model had the best classification results. The classification accuracy on the test set was 86.08%. Edenio Olivares Díaz et al. [38] used logistic regression, a support vector machine model and a partial least squares discriminant analysis model to classify the taste quality of rice, respectively. Optimization was performed on 796 rice samples, 278 samples were tested, and finally, the logistic regression and support vector machine models outperformed the partial least squares model with an accuracy of 94%. Youngwook Seo et al. [39] performed the lossless classification of rice and starch through multiple hyperspectral modes and chemometrics. The highest accuracy achieved was 97.43% through short-wavelength infrared with normalization in the spectral domain. The submission of the developed classification model to the hyperspectral images showed that the fluorescence method achieves the highest accuracy of 81% using LDA. Lu Wang et al. [40] used hyperspectral images to determine the variety and quality of rice. The BPNN model based on data fusion achieved the best results (94.45%), which were superior to the results based on spectral data (89.91%) or image data (88.09%) alone. Zhengjun Qiu et al. [41] used a CNN combined with hyperspectral images to discriminate the varieties of single rice seeds. The classification accuracy of the training and test set was 89.6% and 87.0%, respectively. Shizhuang Weng et al. [42] used a deep learning network with multi-feature fusion combined with hyperspectral images to discriminate the quality of rice; the best result was achieved by PCANet with PCA-processed spectroscopic and texture features with correct classification rates of 98.66% and 98.57% for the training and prediction sets, respectively.

The flavor value of rice is determined by its starch, protein and moisture content. In this paper, the prediction of rice flavor values was performed via hyperspectral imaging combined with the GISPSO-SVM algorithm, and the best discrimination accuracy of 96% was achieved when 50 principal components were retained using the PCA feature algorithm. The flavor value of rice is closely related to its varieties, and there are often some differences between different varieties, but there are also cases where different varieties correspond to the same flavor value, and the determination of rice varieties can discriminate the flavor value of rice to a greater extent, which is similar to the results of this paper.

While the traditional particle swarm optimization (PSO) algorithm is able to quickly find the optimal parameters within the specified search boundaries, it is often plagued by local optima that prevent it from finding the global optimum. This dilemma is further exacerbated by the improper delineation of the parameter search boundaries, which limits the potential of the particles to explore other potentially more optimal solutions, thus significantly diminishing the efficacy of the algorithm. This highlights the importance of establishing the proper parameter search boundaries during the optimization process.

The emergence of the Grid Iterative Search improved PSO (GISPSO) algorithm has facilitated optimization on a global scale. By judiciously determining the parameter search step size during the iteration process, GISPSO is able to narrow down the search boundaries to the neighborhood of the approximation of the global optimum, thus allowing the PSO algorithm to explore this range efficiently. This strategic approach greatly reduces the possibility of convergence of the PSO algorithm to the local optimum and ensures that the global optimum is reached in a time- and effort-saving manner.

In addition, the improved PSO algorithm demonstrated excellent ability in identifying the unique flavors of various rice varieties. By analyzing and comparing the reflectance bands of different rice varieties, the algorithm is able to pinpoint the characteristic wavelengths that exhibit significant differences. The combination of the advantages of a grid search and the PSO algorithm greatly enhances the efficacy of support vector machine (SVM) parameter optimization.

After the spectral preprocessing, feature selection and model training phases, the GISPSO algorithm facilitates the determination of rice flavor values through hyperspectral experiments. The improved accuracy of PSO optimization, especially in the context of extensive and complex datasets, simultaneously alleviates the time requirements associated with a grid search. Nevertheless, the algorithm itself has the potential for further refinement, especially in terms of particle update strategies. The introduction of innovative particle update mechanisms could radically improve the search efficiency of the PSO algorithm.

Although initial preprocessing and feature extraction were performed on the hyperspectral curves, redundant or critical bands may be inadvertently overlooked by different feature selection algorithms during model training. Nonetheless, the accuracy of the flavor value determination was as high as 96%, which underscores the strong confidence in the feasibility of hyperspectral-based flavor analysis of rice. This confirms the optimization capability of grid search iterations within the PSO framework, especially when the number of samples is large or the correlations are complex.

## 4. Conclusions

The quantification of the flavor characteristics of rice using hyperspectral imaging revealed the significant differences in the reflectance at hyperspectral wavelengths among the rice varieties, especially at wavelengths above 450 nm. Beyond this threshold, a unique pattern of reflectance oscillations emerged among the rice varieties. This study culminated in two major discoveries: the accurate prediction of rice flavor profiles using hyperspectral data and improved hyperspectral de-detection techniques to enhance the accuracy of PSO optimization and rice flavor assessment.

The grid-based improved PSO (GISPSO) algorithm combined with feature selection performed slightly better than the GISPSO algorithm that utilizes the full spectral band. The latter method has increased the redundancy due to the large amount of data in the full spectral bands, which affects the PSO algorithm’s pursuit of an optimal solution. In contrast, the GISPSO algorithm combined with feature selection shows stronger applicability for parameter optimization in the case of large datasets and intricate data interrelationships.

The GISPSO algorithm proves to be a valuable tool for validating the effectiveness of the PSO algorithm, especially in cases involving limited data samples and more recognizable data relationships. In cases where the data interrelationships are complex or the PSO algorithm tends to converge to a local optimum, applying the GIS algorithm to perform a global search within the approximation is highly advantageous in guiding the PSO particles towards the realm of global optimization.

This study advances the optimization of the support vector machine (SVM) prediction and optimization algorithms for evaluating the rice flavor profiles through hyperspectral imaging, especially when complex data relationships are involved. It is expected that future developments will further refine the feature selection process, thereby expanding the utility of the method. In addition, more exploration of the GISPSO algorithm is needed, especially for multispectral studies containing large datasets. Follow-up studies will focus on enhancing the selection of regression models, which can help integrate these models into unmanned aerial vehicles (UAVs) for the real-time monitoring of the rice flavor characteristics in agricultural landscapes. In addition, the continued research on the spectral characterization of rice will enrich our understanding of this area in future research efforts.

## Figures and Tables

**Table 1:** RMSE for different regression models and preprocessing approaches.

Model	Original	Smoothing	SNV	MSC
RF	2.04	1.67	1.74	1.8
SVM	1.88	1.56	1.69	1.72
GPR	2.13	1.63	1.71	1.81
KRR	2.06	1.97	1.86	1.75

**Table 2:** Projected results.

Feature Selection	Regression Algorithm	RMSE	Accuracy	Best c	Best g
462bands	SVM	1.59	80%	-	-
	PSO	0.8185	81%	100	12.3227
	GISPSO	0.6736	83%	123.062	9.3279
PCA10	SVM	0.7277	82%	-	-
	PSO	0.3676	92%	9.9879	3.4829
	GISPSO	0.3593	91%	10.3462	3.2641
PCA20	SVM	0.4306	86%	-	-
	PSO	0.4017	87%	13.3521	0.4777
	GISPSO	0.4156	87%	15.0158	0.4566
PCA50	SVM	0.3591	93%	-	-
	PSO	0.3123	93%	23.3732	0.1
	GISPSO	0.3019	96%	420.1067	1.1104
CARS	SVM	0.7892	83%	-	-
	PSO	0.3998	91%	15.4424	3.4181
	GISPSO	0.4033	93%	199.9146	0.0063

**Table 3:** Results for different parameter ranges.

Method	Groups	RMSE	Accuracy	Best c	Best g	Minimum Fitness
PCA50-PSO	1	0.3123	93%	23.3732	0.1	0.00596
	2	0.3509	92%	200	0.2	0.00587
	3	0.3328	91%	298.6688	0.245	0.00572
PCA50-GISPSO	4	0.3019	96%	420.1067	1.1104	0.00568

## Data Availability

Data are contained within the article.
